# Impact of child and parental characteristics on the utilization and outcomes of dental healthcare

**DOI:** 10.3389/froh.2026.1688241

**Published:** 2026-03-20

**Authors:** Regina Mathai, Morgan Santoro, Lisa J. Heaton, Eric P. Tranby, Tamanna Tiwari

**Affiliations:** 1 Department of Pediatric Dentistry, Children’s Hospital Colorado, Aurora, CO, United States; 2 Analytics and Data Insights, CareQuest Institute for Oral Health, Boston, MA, United States; 3 Department of Primary Dental Care, School of Dentistry, University of Minnesota, Minneapolis, MN, United States

**Keywords:** dental caries, dental claims, dental emergency, dental pain, prevention

## Abstract

**Objectives:**

Medical–dental integration promotes early intervention in oral diseases, reduces barriers to care, and optimizes dental home establishment for children. This study aims to explore how child-level [gender, age, number of preventative dental visits (PDVs), and comorbidities] and parent-level [employment, family size, comorbidities, and number of PDVs] characteristics affect dental service claims.

**Methods:**

Merative administrative claims data were analyzed for children aged 0–6 years who had a well-child visit, at least one dental visit between 2019 and 2021, and were covered under private insurance plans (*N* = 198,433). Descriptive statistics and regression analysis were used to predict dental claims, with child- and parent-level factors as predictors.

**Results:**

At the child level, there were higher odds of claims for male children compared with female children for the treatment of dental pain, anesthesia, and emergencies. With increased age, the odds of having a claim for the treatment of dental pain, dental extractions, anesthesia, and emergencies increased. At the parent level, salaried employment (compared with hourly employment) resulted in lower odds of child claims for the treatment of pain extraction, anesthesia, and emergencies.

**Conclusion:**

PDV for children and parental employment decreased the odds of dental service claims, indicating that PDV is valuable for reducing oral health burden.

## Introduction

Well-child visits (WCVs) are essential for delivering preventive healthcare services to the pediatric population in the United States. Many services are included in a WCV, including but not limited to administering immunizations, monitoring developmental milestones, and providing early interventions for behavioral concerns (1). Guidelines for well-child care by the American Academy of Pediatrics (AAP) Council on Pediatric Practice, first published in 1967, recommended 15 well-child visits in the first 3 years of life. Annual visits are recommended from the age of 3 years through adolescence. Since the initial publication of the guidelines, recommendations for services that should be included during the WCV have been changed to include increased screening tests and expanded anticipatory guidance to address topics related to physical and psychosocial health (2).

The American Academy of Family Physicians (AAP) suggests that oral health should be included in the WCV to facilitate proper referral to a dentist (3). Referral to a dentist should occur by 12 months of age, especially for children with significant risk factors, which include inadequate at-home dental care, poor oral hygiene, a mother with dental caries, a diet high in sugar, visible enamel defects on teeth, special healthcare needs, and low socioeconomic status (4). The inclusion of oral health during WCVs does not require much time and can function as an entry point for children into the dental care system (5). In addition, pediatricians have several opportunities to observe infants, toddlers, and school-aged children at regular intervals, including at least 12 WCVs through age three, placing them in a unique position to provide early risk assessment, prevention, detection, and referral (6). In a revised policy statement in 2014, the AAP emphasized medical–dental integration and recommended that pediatricians include anticipatory guidance, conduct dental assessments, and apply fluoride varnish during WCVs. However, one limitation of medical–dental integration is that medical training provides little focus on oral healthcare. Although partnerships exist between medical and dental professionals, there is a need for increased collaboration (7).

Medical–dental integration and the inclusion of preventive dental services in WCVs are effective in reducing caries-related treatment in children. A specific study of a medical office-based preventive dental program, Into the Mouths of Babes (IMB), was conducted using longitudinal claims and enrollment data from North Carolina Medicaid between 2000 and 2006. The IMB program allows children up to 3 years of age to access preventive services at the critical time of primary tooth emergence and the establishment of oral health habits (8). This study concluded that greater than or equal to four IMB visits were effective in reducing the need for future dental treatment. Significant variables associated with a reduction in caries-related treatment (CRTs) included fluoride application at the time of the eruption of the primary tooth and referral to dentists by pediatricians after the detection of the disease.

A cost-effectiveness analysis conducted from the IMB program in North Carolina showed that repeated oral health visits in the primary care setting reduced office visits for dental CRT (9). Although these data constitute only one state and may not be generalizable to the entire population, WCVs provide an opportunity to improve oral health outcomes in children, especially those of lower socioeconomic status and with significant risk factors. This model of medical–dental integration allows pediatricians to play a role in lessening the burden of oral disease and emphasizes a team practice approach between medical and dental professionals.

Moreover, parents' knowledge, behaviors, and socioeconomic status influence children's oral health, with the low socioeconomic status of the family and parents' poor oral health habits contributing to the development of oral diseases. Children from lower socioeconomic classes have the poorest oral health and the highest prevalence and severity of dental caries (10). Barriers to accessing dental services are also associated with increased caries, as these may represent financial hardships that may limit the necessary support required by parents to improve oral health status. In addition, children with increased odds of oral disease are more likely to have parents who also do not regularly visit the dentist (11). Even when adjusting for sociodemographic factors, children are more likely to have a dental visit if their parents also have a dental visit compared with the children of parents who do not have a dental visit. In summary, parents’ oral health-seeking behaviors are likely to have an important effect on their children’s oral health-seeking behaviors. Therefore, efforts to eliminate barriers that target parents and children may assist in improving children's underuse of oral health services (12).

This study aims to explore the relationships between various child- and parent-level characteristics and pediatric dental claims for the treatment of dental pain, extractions, treatment under dental anesthesia, and emergency dental visits.

## Methods

This study was reviewed and it received an exempt status from the WCG IRB. The cohort included claims for children who had a WCV and at least one dental visit between 2019 and 2021. Children aged 0–6 years were included in the dataset. Children who had no dental visits were excluded. Specific health data from reimbursed health claims were used to determine logistic regression models for the outcomes of dental pain claims, extraction claims, anesthesia claims, and emergency visit claims.

### Data source

The Merative (formerly IBM Watson MarketScan) Commercial Claims and Encounters Database was used to create the dataset for this study. This database contains deidentified, patient-specific health data of reimbursed healthcare claims for employees, retirees, and their dependents. The individuals in the database are covered under private insurance plans from over 250 medium- and large-scale employers and health plans. Therefore, this database does not contain Medicaid or Medicare data. Approximately 30 million employees and family members are covered annually in the data.

### Cohort construction

Children aged 0–6 years who were commercially insured and continuously enrolled during the study years were identified to ensure a stable sample with complete claims data. Among these children, those with at least one well-child visit and at least one dental visit were included. Children without any dental claims provide no observable information on dental service use or outcomes; including them would have introduced a large comparison group with structurally zero exposure, reducing comparability and potentially biasing associations toward null findings. The resulting cohort represents a consistently insured pediatric population with documented preventive medical care and observable dental utilization.

### Code definitions

WCVs were defined by ICD-10 diagnosis codes Z00121, Z00129, Z00110, Z00111, Z005, Z0070, Z0071, Z008, Z020, Z021, Z022, Z023, Z024, Z025, Z026, Z0282, and Z0289 and by current procedural terminology (CPT) prevention codes 99381, 99382, 99383, 99384, 99385, 99391, 99392, 99393, 99394, 99395, 99432, and 99461. Dental visits were defined by current dental terminology (CDT) codes starting with D11–D19. Treatments for dental pain were defined by CDT codes D0140 (limited oral evaluation-problem focused) and D9110 (palliative treatment of dental pain-per visit). D0140 is defined as a focused evaluation for a specific problem or concern, commonly used for urgent presentations, while D9110 denotes non-definitive treatment provided to relieve dental pain during a visit. Extractions were defined by CDT codes D7111, D7210, D7140, and D7250. Treatment under general anesthesia was defined by CDT codes D9222, D9223, D9239, D9243, and D9248. Emergency visit claims were defined by CDT codes D0170, D0171, D0190, D0191, D9311, and D7111.

### Variables

The data included child- and parent-level variables. At the child level, the variables included age, gender, and number of dental claims for preventive, extraction, anesthesia, and emergency visit claims. At the parent level, the variables included parents' employment status, family size, and the number of parents' dental preventive visit claims.

### Data analysis

**Figure 1:**
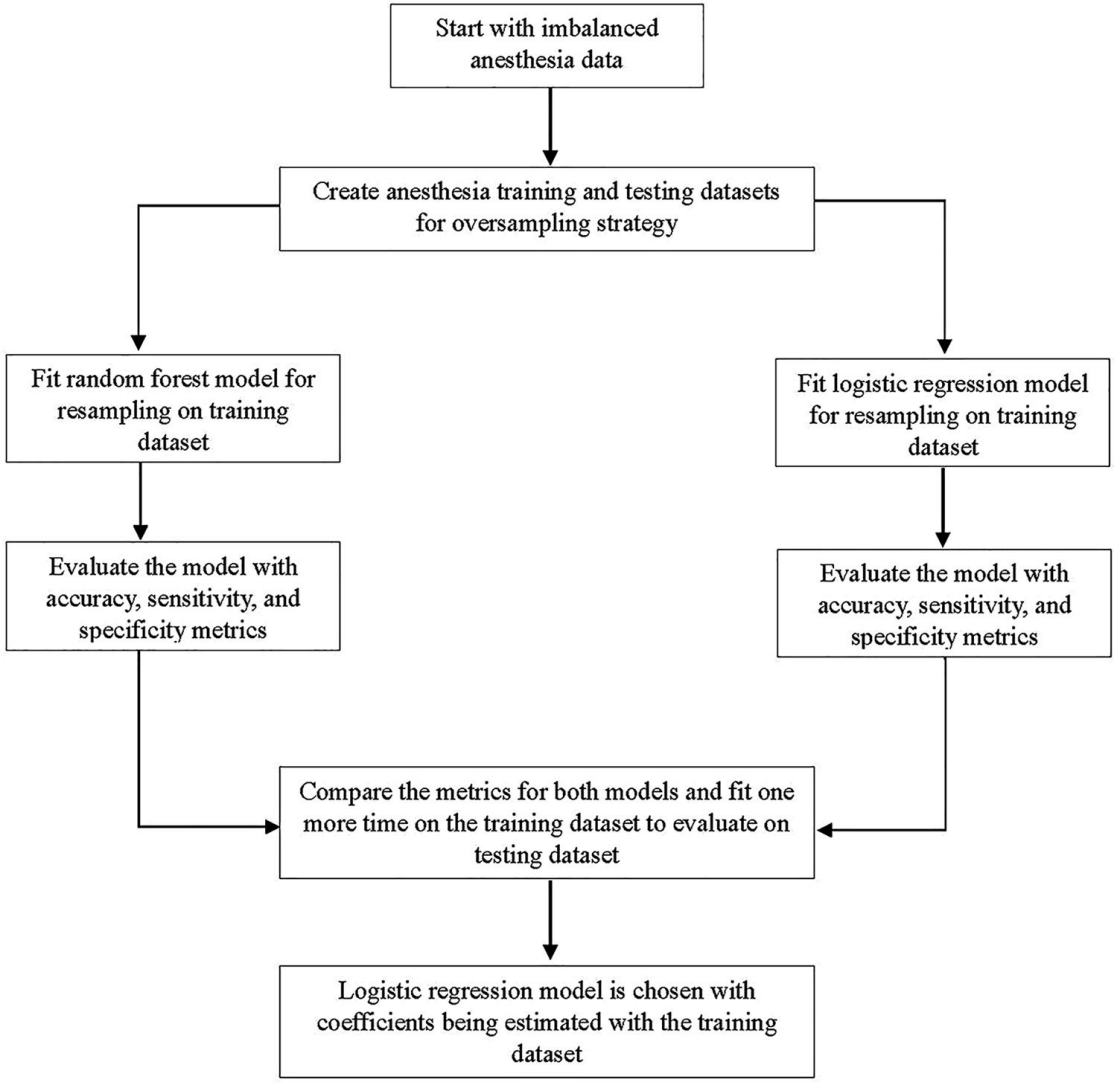
A study flowchart describing oversampling via SMOTE (synthetic minority oversampling technique).

Descriptive and regression analyses were used to evaluate different models. Our modeling goal was to predict one or more anesthesia claims and one or more emergency department claims in children aged 6 years and younger. However, for each of these classes, there was an uneven distribution of observations. For example, there were 193,197 children under the age of six without anesthesia claims, compared with only 5,236 children with anesthesia claims. Because of the small sample size of children with anesthesia claims, the different types of anesthesia (e.g., general anesthesia, IV anesthesia, and non-IV conscious sedation) were consolidated for the analysis. The subsampling technique SMOTE (Synthetic Minority Oversampling Technique) was used to balance class distribution in these variables (Figure 1) (13). For both variables, a logistic regression model and a random forest model were built and evaluated using resampling. These models were then compared with each other. For anesthesia claims, accuracy (0.62), sensitivity (0.65), and specificity (0.60) were moderate but mostly consistent. For the random forest model, accuracy (0.81) and sensitivity (0.81) were high, but specificity was very low (0.31). Therefore, the logistic regression model was chosen. Also for emergency claims, the logistic regression model was chosen. The logistic regression model also showed moderate accuracy (0.62), sensitivity (0.62), and specificity (0.60). However, in the random forest model, accuracy (0.81) and sensitivity (0.82) were high, but specificity (0.31) was very low. Once the model evaluation was completed, the odds ratio coefficients were estimated and visualized.

## Results

Table 1 presents the demographic characteristics of claims in the sample (*N* = 198,433). The table presents the number and percentage of claims for dental pain, extraction, dental anesthesia, and dental emergencies. These variables were stratified by child age, gender, and the number of dental preventive visits. In addition, Table 2 provides parental characteristics such as employment status, family size, and parental dental preventive visits.

**Table 1:** Child-level demographic characteristics of the sample (*N* = 198,433).

Variable	Claims				
	Total	Dental pain	Extraction	Dental anesthesia	Dental emergency
	*N* (% of dental claims)	*N* (% of claims within-variable level)	*N* (% of claims within-variable level)	*N* (% of claims within-variable level)	*N* (% of claims within-variable level)
Child's gender					
Female	97,166 (49.0)	13,920 (14.3)	8,885 (9.1)	2,464 (2.5)	1,475 (1.5)
Male	101,267 (51.0)	14,892 (14.7)	9,445 (9.3)	2,772 (2.7)	1,495 (1.5)
Child's age (years)					
0	261 (0.1)	21 (8.0)	2 (0.8)	1 (0.4)	1 (0.4)
1	1,862 (0.9)	208 (11.2)	33 (1.8)	7 (0.4)	23 (1.2)
2	13,820 (7.0)	1,286 (9.3)	206 (1.5)	96 (0.7)	161 (1.2)
3	21,822 (11.0)	2,200 (10.1)	474 (2.2)	259 (1.2)	272 (1.2)
4	31,852 (16.1)	3,783 (11.9)	1,329 (4.2)	743 (2.3)	405 (1.3)
5	46,552 (23.5)	6,936 (14.9)	4,181 (9.0)	1,553 (3.3)	619 (1.3)
6	82,264 (41.5)	14,378 (17.5)	12,105 (14.7)	2,577 (3.1)	1,489 (1.8)
Child’s dental prevention visits					
None	4,941 (2.5)	1,336 (27.0)	310 (6.3)	70 (1.4)	264 (5.3)
1	29,331 (14.8)	2,312 (7.9)	1,146 (3.9)	390 (1.3)	266 (0.9)
2	29,840 (15.0)	3,217 (10.8)	1,904 (6.4)	621 (2.1)	293 (1.0)
3	31,011 (15.6)	4,018 (13.0)	2,530 (8.2)	772 (2.5)	391 (1.3)
4	33,558 (16.9)	4,986 (14.9)	3,224 (9.6)	950 (2.8)	496 (1.5)
5	39,807 (20.1)	6,142 (15.4)	3,933 (9.9)	1,159 (2.9)	589 (1.5)
6+	29,945 (15.1)	6,801 (22.7)	5,283 (17.6)	1,274 (4.3)	671 (2.2)

For variables where the total does not add up to the whole *N* (198,433), this discrepancy results from the null (“N/A”) values in the dataset.

**Table 2:** Parent-level demographic characteristics of the sample (*N* = 198,433).

Variable	Claims				
	Total	Dental pain	Extraction	Dental anesthesia	Emergency
Parent’s employment status					
Hourly	55,264 (27.9)	8,531 (15.4)	6,098 (11.0)	2,176 (3.9)	965 (1.7)
Salaried	109,974 (55.4)	15,278 (13.9)	9,279 (8.4)	2,313 (2.1)	1,564 (1.4)
Unknown	33,173 (16.7)	4,997 (15.1)	2,951 (8.9)	746 (2.2)	441 (1.3)
Family size					
2	9,090 (4.8)	1,545 (17.0)	1,022 (11.2)	314 (3.5)	195 (2.1)
3	33,279 (17.6)	4,990 (15.0)	2,930 (8.8)	809 (2.4)	517 (1.6)
4	84,644 (44.8)	12,129 (14.3)	7,273 (8.6)	2,023 (2.4)	1,217 (1.4)
5	44,960 (23.8)	6,353 (14.1)	4,262 (9.5)	1,239 (2.8)	647 (1.4)
6+	17,166 (9.1)	2,434 (14.2)	1,756 (10.2)	525 (3.1)	247 (1.4)
Parent’s dental prevention visits					
None	53,412 (26.9)	8,190 (15.3)	6,106 (11.4)	1,854 (3.5)	819 (1.5)
1	23,877 (12.0)	3,548 (14.9)	2,476 (10.4)	698 (2.9)	372 (1.6)
2	22,096 (11.1)	3,127 (14.2)	1,956 (8.9)	580 (2.6)	324 (1.5)
3	21,299 (10.7)	3,010 (14.1)	1,744 (8.2)	442 (2.1)	287 (1.3)
4	24,224 (12.2)	3,412 (14.1)	1,866 (7.7)	528 (2.2)	382 (1.6)
5	33,550 (16.9)	4,686 (14.0)	2,623 (7.8)	692 (2.1)	469 (1.4)
6+	19,953 (10.1)	2,833 (14.2)	1,557 (7.8)	441 (2.2)	317 (1.6)

s.d., standard deviation.

For variables where the total does not add up to the whole *N* (198,433), this discrepancy is caused by the null (“N/A”) values in the dataset.

Table 3 presents the logistic regression analysis results predicting claims for children's treatment of dental pain. Male children had higher odds of pain claims compared with female children (OR = 1.04; CI, 1.01, 1.07, *p* < 0.05). As the age of the child increased, the odds of dental pain claims also increased (OR = 1.14; 95% CI = 1.13, 1.16; *p* < 0.001). Children who had at least one preventive visit had lower odds of having a claim for dental pain treatment compared with those without a preventive visit. However, as the number of preventive claims increased, so did the odds of visiting for the treatment of dental pain. For example, compared with children without a preventive visit, those with one preventive visit had 79% less chance of having a dental pain claim (OR = 0.21; 0.19, 0.23; *p* < 0.001), while this chance decreased to 34% for children with six or more preventive visits (OR = 0.66; 0.61, 0.71; *p* < 0.01). Table 3 also provides an analysis of parent-level factors for child pain claims. Compared with children whose parents had an hourly income, children whose parents had a salaried income had lower odds of receiving a treatment for dental pain (OR = 0.87; 95% CI = 0.85, 0.90, *p* < 0.001). In addition, children from a family size of greater than 2 (family sizes 3, 4, 5, and 6 or greater) showed lower odds of pain claims. Compared with a family size of 2, children from a family size of greater than 3 had a 13% lower chance of child pain claims, and this chance reduced as the family size increased (family size of 4, 20% lower chance; family size of 5, 23% lower chance; and family size of 6 or more, 22% lower chance, *p* < 0.001). There was no significant difference in the odds of a child’s dental pain claim for those whose parents had one PVD (OR = 0.98; 95% CI = 0.94, 1.02, *p* = 0.4), but as the number of parental PVDs increased, the odds of children's dental pain claims decreased.

**Table 3:** Logistic regression analyses predicting pediatric claims for treatment of dental pain.

Variable	Odds ratio	95% confidence interval	*P*-value
Child's gender			
Female	Ref	Ref	Ref
Male	1.04	1.01, 1.07	**0.004**
Child's mean (s.d.) age	1.14	1.13, 1.16	**<0.001**
Number of child’s dental prevention visits			
None	Ref	Ref	Ref
1	0.21	0.19, 0.23	**<0.001**
2	0.29	0.27, 0.31	**<0.001**
3	0.34	0.32, 0.37	**<0.001**
4	0.40	0.37, 0.43	**<0.001**
5	0.41	0.38, 0.45	**<0.001**
6+	0.66	0.61, 0.71	**<0.001**
Parent’s employment status			
Hourly	Ref	Ref	Ref
Salaried	0.87	0.85, 0.90	**<0.001**
Unknown	0.96	0.92, 1.00	**0.03**
Family size			
2	Ref	Ref	Ref
3	0.87	0.82, 0.93	**<0.001**
4	0.80	0.75, 0.85	**<0.001**
5	0.77	0.73, 0.82	**<0.001**
6+	0.78	0.73, 0.84	**<0.001**
Number of parent’s dental prevention visits			
None	Ref	Ref	Ref
1	0.98	0.94, 1.02	0.4
2	0.90	0.86, 0.95	**<0.001**
3	0.88	0.84, 0.93	**<0.001**
4	0.84	0.80, 0.88	**<0.001**
5	0.76	0.73, 0.79	**<0.001**
6+	0.73	0.70, 0.77	**<0.001**

Ref, reference value.

Values in bold indicate   *p* ≤ 0.05.

Table 4 describes the logistic regression analysis results predicting claims for children's extractions. There was no significant difference by gender in predicting extractions (OR = 1.03; 95% CI = 1.00, 1.06; *p* = 0.07). As the age of the child increased, the odds of extraction claims also increased (OR = 1.78; 95% CI = 1.74, 1.81; *p* < 0.001). There was a progressive positive correlation between dental preventive visits and extraction claims. As the number of dental preventive visits increased from 1 to 6 or more, compared with no dental preventive visit, the odds of extraction moved from 59% decreased odds for 1 dental preventive visit to 1.5 times higher odds of extraction claims for six or more dental preventive visits (*p*'s < 0.001). Children whose parents had salaried employment had decreased odds of having an extraction claim compared with those whose parents reported hourly employment (OR = 0.74; 95% CI = 0.71, 0.77, *p* < 0.001). Compared with a family size of two, children from a family size of greater than 3 had a 19% lower chance of child pain claims, and the chance of a pain claim remained reduced as the family size increased (family size of 4, 27% lower chance; family size of 5, 21% lower chance; and family size of 6 or more, 14% lower chance, *p* < 0.001). Children whose parents had one or more preventive dental visits had significantly lower odds of having extraction claims than those whose parents had no preventive visits. Children whose parents had one dental visit had 7% (*p* < 0.004) lower chance of having an extraction claim, and the chances reduced as the parental dental visits increased; for six or more parental dental visits, the child's extraction claims were less than 52% (*p* < 0.001).

**Table 4:** Logistic regression analyses predicting pediatric claims for extraction.

Variable	Odds ratio	95% confidence interval	*P*-value
Child's gender			
Female	Ref	Ref	Ref
Male	1.03	1.00,1.06	0.07
Child's mean (s.d.) age	1.78	1.74,1.81	**<0.001**
Number of child’s dental prevention visits			
None	Ref	Ref	Ref
1	0.41	0.36, 0.48	**<0.001**
2	0.63	0.55, 0.72	**<0.001**
3	0.74	0.65, 0.85	**<0.001**
4	0.82	0.72, 0.94	**<0.001**
5	0.81	0.71, 0.92	**<0.001**
6+	1.51	1.32, 1.72	**<0.001**
Parent’s employment status			
Hourly	Ref	Ref	Ref
Salaried	0.74	0.71, 0.77	**<0.001**
Unknown	0.80	0.76, 0.84	**<0.001**
Family size			
2	Ref	Ref	Ref
3	0.81	0.75, 0.87	**<0.001**
4	0.73	0.68, 0.78	**<0.001**
5	0.79	0.73, 0.85	**<0.001**
6+	0.86	0.79, 0.94	**<0.001**
Number of parent’s dental prevention visits			
None	Ref	Ref	Ref
1	0.93	0.88, 0.98	**0.004**
2	0.74	0.70, 0.79	**<0.001**
3	0.67	0.63, 0.71	**<0.001**
4	0.59	0.55, 0.62	**<0.001**
5	0.53	0.50, 0.56	**<0.001**
6+	0.48	0.45, 0.52	**<0.001**

Ref, reference value.

Values in bold indicate  *p* ≤ 0.05.

Table 5 provides details of the analysis of claims for procedures completed under anesthesia for children. Male children had higher odds of anesthesia claims compared with their female counterparts (OR = 1.10; 95% CI = 1.09, 1.12; *p* < 0.001). As the age of the child increased, the odds of anesthesia claims also increased (OR = 1.28; 95% CI = 1.27, 1.28; *p* < 0.001). Compared with children without a preventive visit, those with one preventive visit had lower odds of having a dental anesthesia claim (OR = 0.89; 95% CI = 0.83, 0.96; *p* < 0.001). Meanwhile, having two or more preventive visits was associated with higher odds of having dental claim visits. For example, children who had six or more preventive visits had 3.29 times higher odds of having a dental anesthesia claim than children without a preventive visit (OR = 3.29; 95% CI = 3.07, 3.52; *p* < 0.001).

**Table 5:** Logistic regression analyses predicting pediatric claims for treatment under dental anesthesia.

Variable	Odds ratio	95% confidence interval	*P*-value
Child's gender			
Female	Ref	Ref	Ref
Male	1.10	1.09, 1.12	**<0.001**
Child's mean (s.d.) age	1.28	1.27, 1.28	**<0.001**
Number of child’s dental prevention visits			
None	Ref	Ref	Ref
1	0.89	0.83, 0.96	**<0.001**
2	1.40	1.31, 1.50	**<0.001**
3	1.67	1.57, 1.80	**<0.001**
4	1.96	1.83, 2.09	**<0.001**
5	2.16	2.02, 2.31	**<0.001**
6+	3.29	3.07, 3.52	**<0.001**
Parent’s employment status			
Hourly	Ref	Ref	Ref
Salaried	0.55	0.54, 0.56	**<0.001**
Unknown	0.56	0.55, 0.58	**<0.001**
Family size			
2	Ref	Ref	Ref
3	0.83	0.80, 0.87	**<0.001**
4	0.89	0.86, 0.93	**<0.001**
5	0.94	0.90, 0.97	**<0.001**
6+	0.93	0.89, 0.97	**<0.001**
Number of parent’s dental prevention visits			
None	Ref	Ref	Ref
1	0.62	0.60, 0.63	**<0.001**
2	0.53	0.51, 0.54	**<0.001**
3	0.39	0.38, 0.40	**<0.001**
4	0.38	0.37, 0.40	**<0.001**
5	0.36	0.35, 0.37	**<0.001**
6+	0.36	0.35, 0.74	**<0.001**

Ref, reference value.

Values in bold indicate *p* ≤ 0.05.

Table 5 also shows that parents' salaried employment is a protective factor for children's anesthesia claims compared with parents who have hourly employment (OR = 0.55; 95% CI = 0.54, 0.56; *p* < 0.001). In addition, a smaller family size was more protective of children's anesthesia claims. Compared with children from two-person families, children from three-person families had lower odds of a dental anesthesia claim (OR = 0.83; 95% CI = 0.80, 0.87; *p* < 0.001); the protective factor slightly decreased for children from six-person families (OR = 0.93; 95% CI = 0.89, 0.97; *p* < 0.001). As the number of parents' prevention visits increased, the odds of children's dental anesthesia claims decreased. Compared with children whose parents did not have any preventive visit, those whose parents had one preventive visit had lower odds of a dental anesthesia claim (OR = 0.62; CI = 0.60, 0.63; *p* < 0.001), and children whose parents had six or more preventive visits had even lower odds of a dental anesthesia claim (OR = 0.36; CI = 0.35, 0.74; *p* < 0.001).

Table 6 presents the logistic regression analyses predicting claims for children's emergency dental visits. Male children had decreased odds of having one or more emergency claims compared with female children (OR = 0.95; 95% CI = 0.94–0.97; *p* < 0.001). As the age of the child increased, the odds of having an emergency visit claim increased (OR = 1.22; 95% CI = 1.21, 2.23; *p* < 0.001). Children with at least one preventive visit had lower odds of having emergency visits than those without a preventive visit. The protective factor was higher for one preventive visit (OR = 0.10; 95% CI = 0.09, 0.10; *p* < 0.001) and reduced for six or more preventive visits (OR = 0.22; 95% CI = 0.21, 0.23; *p* < 0.001). In addition, Table 6 shows that the salaried income of parents is a protective factor, with salaried income showing decreased odds of dental emergency claims (OR = 0.83; 95% CI = 0.82, 0.85; *p* < 0.000). Compared with children from two-person families, those from three-person families had lower odds of emergency claims (OR = 0.72; 95% CI = 0.69, 0.75; *p* < 0.001), and the protective factor increased for children from 6-person families (OR = 0.60; 95% CI = 0.56, 0.63; *p* < 0.001). Similarly, parental preventive dental claims were also a protective factor with an increase in preventive claims for the parent showing decreased odds of emergency dental claims with increasing numbers of parental PVDs (*p*s < 0.05).

**Table 6:** Logistic regression analyses predicting pediatric claims for emergency dental visits.

Variable	Odds ratio	95% confidence interval	*P*-value
Child's gender			
Female	Ref	Ref	Ref
Male	0.95	0.94, 0.97	**<0.001**
Child's mean (s.d.) age	1.22	1.21, 2.23	**<0.001**
Number of child’s dental prevention visits			
None	Ref	Ref	Ref
1	0.10	0.09, 0.10	**<0.001**
2	0.10	0.10, 0.11	**<0.001**
3	0.12	0.12, 0.13	**<0.001**
4	0.14	0.14, 0.15	**<0.001**
5	0.14	014, 0.15	**<0.001**
6+	0.22	0.21, 0.23	**<0.001**
Parent’s employment status			
Hourly	Ref	Ref	Ref
Salaried	0.83	0.82, 0.85	**<0.001**
Unknown	0.72	0.70, 0.74	**<0.001**
Family size			
2	Ref	Ref	Ref
3	0.72	0.69, 0.75	**<0.001**
4	0.70	0.67, 0.73	**<0.001**
5	0.65	0.63, 068	**<0.001**
6+	0.60	0.56, 0.63	**<0.001**
Number of parent’s dental prevention visits			
None	Ref	Ref	Ref
1	0.73	0.72, 0.75	**<0.001**
2	0.67	0.65, 0.69	**<0.001**
3	0.62	0.60, 0.64	**<0.001**
4	0.73	0.71, 0.75	**<0.001**
5	0.63	0.61, 0.64	**<0.001**
6+	0.66	0.64, 0.68	**<0.001**

Ref, reference value.

Values in bold indicate  *p* ≤0.05.

## Discussion

To the best of our knowledge, this is the first study to explore the interplay of child- and parent-level characteristics that affect dental healthcare utilization and outcomes in children. This study examines characteristics at the child level, including gender, age, and number of preventative dental visit (PDV), and how these factors affect dental claims for pain, extractions, anesthesia, and emergencies. The study also explores the relationship between dental claims for children based on parental characteristics, including employment, family size, and number of parental PDV.

The results of this study revealed significant trends. First, male children had higher odds of dental pain, anesthesia, and emergency claims. Female children exhibited more favorable oral health habits compared with male children, including brushing their teeth more regularly (14). This is possibly because female children may be more likely to imitate their mothers' behavior, while male children are more likely to imitate the behavior of their fathers (10). Second, older children had higher odds of dental pain, anesthesia, dental extraction, and dental emergency claims than younger children. As age increases, dentition changes to include more teeth. In addition, as age increases, the number of dental visits also increases, allowing for more reported claims (15).

Third, more PDVs— both for children and for their parents—decreased the odds of children undergoing treatment for dental pain, extraction, dental anesthesia, and emergency dental visits. This coincides with the notion that prevention decreases disease initiation and development. When one parent’s prevention claim was analyzed with anesthesia claims, there was a 38% decrease in anesthesia claims for the child compared with no parent preventive visits. In addition, when six parent prevention claims were analyzed with anesthesia claims, there was a 64% decrease in anesthesia claims for children. Furthermore, the odds of anesthesia claims for children continued to decrease; as parental preventive claims increased by two, three, four, five, and six, the dental anesthesia claims for children decreased (43.6%, 62%, 63.6%, 63%, and 63.2%).

The American Academy of Pediatrics and the American Academy of Pediatric Dentistry (AAPD) recommend the inclusion of the first caries risk assessment by child health professionals at 6 months of age during WCVs and the establishment of a dental home by the age of one (16). Tiwari et al. showed an overall trend of a higher number of preventive dental visits for all children who had a prior WCV (17). Collaborative relationships between pediatricians and dentists allow for the identification of high-risk children, early utilization of preventive dental services, and a decrease in dental disease (16). Moreover, medical–dental integration with the colocation of services in a hospital setting can increase the utilization of preventive services and decrease burden. According to Tiwari et al., the most utilized location for WCVs is a hospital, and the majority of children in the United States, regardless of insurance, are seen at office or outpatient hospital-based locations (18).

Lastly, parental employment, as defined by salaried income, lowered the odds of dental pain, dental extraction, anesthesia, and emergency claims. Salaried income compared with parental hourly income was a protective factor and had a 26% lower chance of child extraction claims. Salaried income also had a 17% decrease in dental emergency claims when compared with hourly employment. This trend is consistent with that in the existing literature. Family income and parental education levels are significant predictors of children's oral health–related quality of life (19). In addition, children from families with insufficient income visited dentists more frequently for dental treatment. Parents with a high educational level and sufficient income were more attentive to preventive factors related to their children's oral health than those with a low educational level and insufficient income (20).

This study used claims data, specifically for children covered by private insurance plans; thus, certain limitations exist. For inclusion in the study, children needed to have at least one WCV, and the study excluded children who had no dental visits. Also, the children included in this study had private insurance. Thus, the results are pertinent to a specific population of children and cannot be generalized to a larger population. For further research, children covered under state and federal plans should be included in the analysis. Some procedure types had small sample sizes, such as dental anesthesia, which prevented further analysis of various types of anesthesia. It would be beneficial and potentially clarifying for future research to focus on the individual types of dental anesthesia. Importantly, claims data do not confirm clinical diagnoses and do not contain individual-level clinical information; they capture billed procedures rather than underlying clinical conditions or disease severity. In addition, as diagnostic codes are still not used in dentistry, it is difficult to extrapolate the baseline clinical condition being treated; accordingly, procedure codes serve as proxies for clinical indications (e.g., “emergency,” treatment for dental pain). For example, the use of D0140 and D9110 to indicate “dental pain/urgent encounters” may misclassify some visits (e.g., problem-focused evaluations for issues other than pain or palliative care for irritation rather than pain). Overall, this dataset provides information on the types of procedures performed but not confirmed diagnoses.

In conclusion, this study established a trend of decreased dental service claims for children with a PDV, indicating that a PDV is valuable in reducing the oral health burden of the disease. In addition, parental employment is a protective factor for children, resulting in lower odds of dental service claims. This study reiterates that WCVs should continue to include preventive dental services and provide proper referrals to dentists. Further research should target preventive programs and policies to expand population-specific interventions that decrease the burden of oral disease.

## Data Availability

The raw data supporting the conclusions of this article will be made available by the authors, without undue reservation.
